# Physical Exercise and Mobile Social Media Dependence Among University Students: Exploratory Conditional-Process Patterns Involving Loneliness and Self-Control

**DOI:** 10.3390/bs16101908

**Published:** 2026-10-15

**Authors:** Xiaojin Wang, Xiaoyu Wu, Jin Yang, Fenghua Zhang

**Affiliations:** 1 School of Psychology, Jiangxi Normal University, Nanchang 330022, China; 202350000022@jxnu.edu.cn; 2 School of Humanities, Jiangxi University of Chinese Medicine, Nanchang 330004, China; 202101037002@jxutcm.edu.cn

**Keywords:** physical exercise, mobile social media dependence, loneliness, self-control, university students, discriminant validity

## Abstract

Physical exercise may be associated with less problematic digital use, but the evidence is heterogeneous, and the interpersonal and self-regulatory correlates of this association remain uncertain. We examined the observed-score relationships among exercise, loneliness, self-control, and mobile social media dependence in 205 non-physical-education undergraduates in Jiangxi, China. Item-level questionnaire data were rescored using published keys. Exercise was associated with lower dependence (r = −0.823), lower loneliness (r = −0.799), and higher self-control (r = 0.723; all *p* < 0.001). However, loneliness and dependence remained extremely highly correlated, and a targeted diagnostic CFA estimated a boundary-level latent correlation of approximately 0.999, indicating inadequate discriminant validity. An exploratory PROCESS-equivalent Model 15 showed significant exercise × self-control (B = 0.0133, *p* < 0.001) and loneliness × self-control (B = 1.0042, *p* < 0.001) interactions; the conditional product-of-coefficients index was −0.0177, 95% bootstrap CI [−0.0244, −0.0113]. HC3 and influential-case sensitivity analyses preserved the interaction pattern. The data therefore support a robust observed-score association between greater exercise and lower dependence, but they do not establish a distinct loneliness-mediated mechanism or causal ordering. These findings highlight the importance of integrating behavioral, interpersonal, and self-regulatory perspectives while prioritizing construct differentiation and measurement validity in research on university students’ digital well-being.

## 1. Introduction

University students increasingly use mobile social media for academic coordination, friendship maintenance, leisure, and emotion regulation. The central behavioral-health question is therefore not whether social media is uniformly beneficial or harmful, but when routine use becomes difficult to regulate and begins to interfere with daily functioning (Andreassen, 2015; Cheng et al., 2026; Elhai et al., 2017; Fassi et al., 2025; Marciano et al., 2021; Orben et al., 2022; Valkenburg et al., 2022). In this study, mobile social media dependence denotes a behavioral-risk pattern characterized by salience, compulsive engagement, withdrawal, and conflict rather than a clinical diagnosis. Because the university period combines expanding autonomy with strong peer-belonging and self-management demands, identifying modifiable correlates of problematic mobile social media use remains relevant to digital well-being.

Physical exercise is one candidate behavioral resource. Large observational and meta-analytic studies link physical activity with better mental health, although these findings do not establish an effect on mobile social media dependence (Chekroud et al., 2018; Rebar et al., 2015). Exercise is planned and structured, and repetitive activity is undertaken to maintain or improve fitness (Caspersen et al., 1985), whereas physical activity is the broader category of bodily movement considered in public-health recommendations (World Health Organization, 2020). The present study assesses exercise specifically with the PARS-3 exercise-quantity index; accordingly, “exercise” is used for the focal study variable, whereas “physical activity” is retained when summarizing studies that used broader activity measures. Exercise may structure time, provide non-digital reward, support affect regulation, and, when performed socially, create opportunities for face-to-face contact. These processes are plausible correlates rather than assumed causal mechanisms in the present cross-sectional design.

Evidence linking exercise or physical activity with problematic digital use is suggestive but not uniform. Systematic reviews of university-student studies generally report an inverse association, although effect sizes and methods vary, and the evidence remains predominantly cross-sectional (Pirwani & Szabo, 2024, 2025). A randomized trial in college students also reported reductions in problematic smartphone use after a 12-week mind–body exercise program (Lu et al., 2020), although its multicomponent intervention does not isolate exercise from other therapeutic ingredients. Recent longitudinal evidence suggests that within-person elevations in self-reported exercise-adherence tendency can precede lower problematic smartphone use, but causal prevention still cannot be inferred (Gai et al., 2026). Conversely, other university samples have found no meaningful association between physical activity and smartphone-addiction status or screen time (Kumban et al., 2025). Thus, the relevant gap is not simply whether exercise and problematic digital use covary, but under what measurement and contextual conditions the association appears, and which interpersonal or self-regulatory correlates accompany it.

Loneliness was considered as a candidate interpersonal correlate, but the literature does not justify assuming that exercise necessarily reduces loneliness. Loneliness reflects perceived inadequacy in the quantity or quality of social relationships (Cacioppo et al., 2006). Compensatory internet-use accounts propose that unmet offline relational needs can motivate online reassurance seeking; persistent or predominantly passive online engagement may in turn intensify social comparison or displace richer in-person interaction (Nowland et al., 2018; Primack et al., 2017). Evidence for social displacement remains limited, however, and increased social media use cannot generally be assumed to replace face-to-face contact (Hall & Liu, 2022). A short randomized trial in undergraduates found lower loneliness following a reduction in use of three social media platforms (Hunt et al., 2018), suggesting that some effects depend on how use is modified. Reviews of physical activity and loneliness report mixed and heterogeneous findings, with social context appearing important: group-based activity, coaching, and deliberate social-support components may be more relevant to loneliness than activity performed alone (Ahn et al., 2024; Pels & Kleinert, 2016). Recent meta-analytic evidence also indicates that group-based physical-activity formats do not uniformly outperform individual formats on psychosocial outcomes, underscoring the importance of context rather than assuming an inherent social benefit of exercise (Kritz et al., 2026). Daily-life research in young adults further links physical activity with less negative affect during periods without social contact, but this compensatory pattern does not itself demonstrate reduced loneliness (Benedyk et al., 2024). We therefore treat the exercise–loneliness link as an empirical association to be tested, not as a universal or China-specific effect.

Self-control provides a more established self-regulatory correlate. It refers to the capacity to regulate impulses, attention, emotion, and behavior in the service of longer-term goals (Baumeister, 2002; Tangney et al., 2004). Meta-analytic and prospective cohort studies further support its relevance across behavioral and health outcomes (de Ridder et al., 2012; Moffitt et al., 2011). Contemporary accounts emphasize that successful self-control depends not only on a stable disposition but also on how people structure situations and manage temptation (Duckworth et al., 2016). Lower self-control has been associated with both problematic internet use and loneliness in college students (Fan et al., 2022), and longitudinal/personality evidence has also linked lower self-control with subsequent loneliness (Stavrova et al., 2022). These findings make it plausible that self-control could alter how strongly loneliness co-occurs with compensatory or difficult-to-regulate online engagement. Nevertheless, because moderation of the loneliness–dependence association has less direct prior evidence than the main self-control associations, this component of the present model is treated as theoretically motivated but exploratory.

The study addresses two specific gaps. First, previous university-student research has often positioned self-control as a mediator between exercise and problematic internet/mobile-phone use or has placed loneliness in a chain mediation role (Xu & Tang, 2024; Zhong et al., 2021), leaving less evidence on whether self-control changes the strength of the exercise–dependence and loneliness–dependence associations. An adolescent study instead examined depression as a mediator and difficulty describing feelings as a moderator of the physical activity–social media dependence association (W. Wang et al., 2025), indicating that existing conditional models differ in both population and proposed correlates. Second, conditional-process studies in this area rarely make construct differentiation a prerequisite for interpreting indirect pathways. We therefore evaluated an a priori architecture in which exercise was the behavioral exposure, loneliness the candidate interpersonal correlate, and self-control a moderator of the direct exercise–dependence and loneliness–dependence associations, while explicitly testing discriminant validity before drawing construct-level conclusions. This measurement-first approach is central to the study because an indirect-effect interpretation is only meaningful if the proposed mediator, moderator, and outcome can be empirically distinguished.

**Figure 1:**
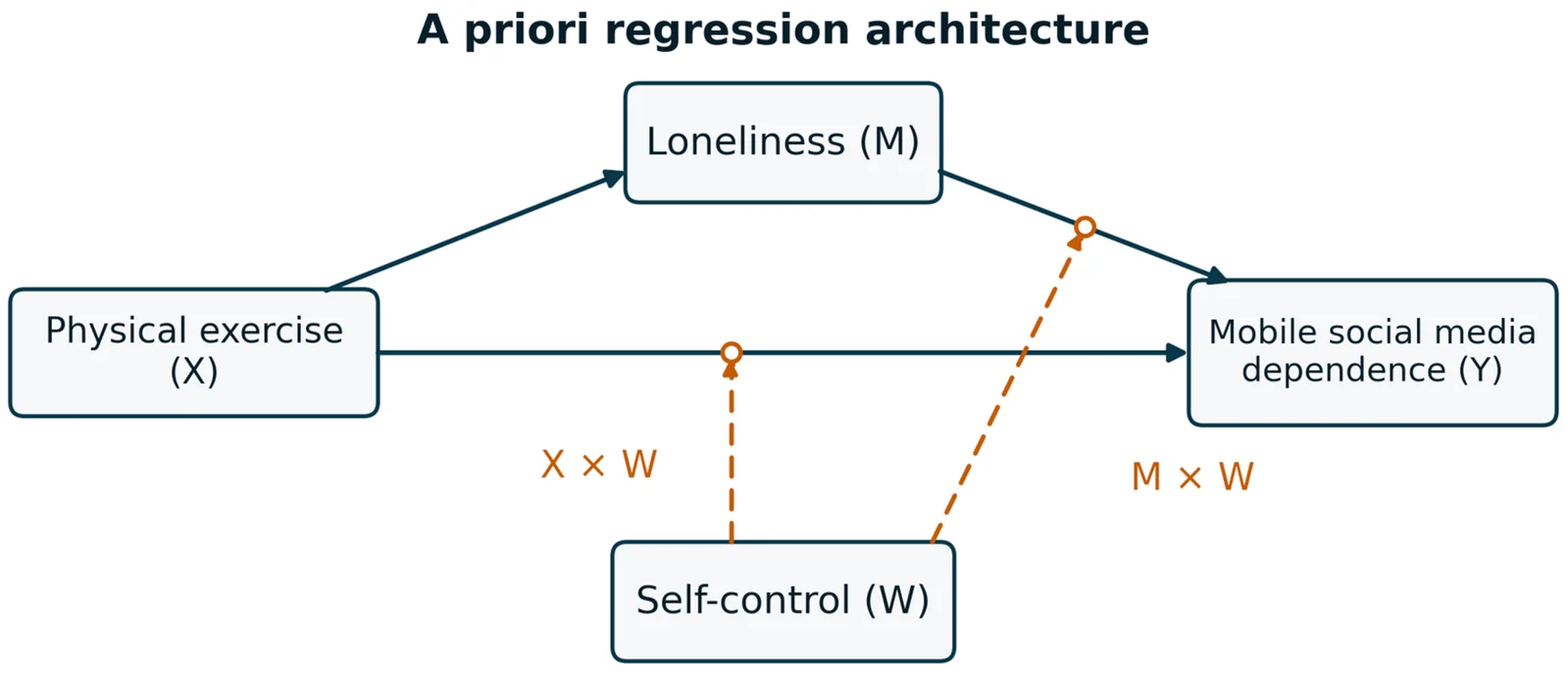
A priori regression architecture. Dashed arrows identify the exercise × self-control and loneliness × self-control terms in the dependence equation. The loneliness equation includes exercise only. Arrows specify regression associations and do not establish causal ordering. Construct-level mediation interpretation was contingent on adequate discriminant validity.

Four a priori hypotheses motivated the analyses: H1, physical exercise is negatively associated with mobile social media dependence; H2, loneliness statistically carries an indirect negative association between exercise and dependence; H3, self-control moderates the direct exercise–dependence association; and H4, self-control moderates the loneliness–dependence association, thereby conditioning the product-of-coefficients association through loneliness. Importantly, H2 and H4 were specified at the construct level and were considered interpretable as mediation/moderated mediation only if the measurement diagnostics supported adequate construct differentiation. When that condition was not met, the corresponding analyses were retained for transparency but interpreted as exploratory observed-score associations rather than confirmation of a distinct psychological mechanism. Figure 1 presents the a priori regression architecture.

## 2. Materials and Methods

### 2.1. Participants and Procedure

The analytic sample comprised 205 non-physical-education undergraduates from three universities in Jiangxi Province, China. The source recruitment plan used strata defined by institutional type (teacher-training, medical, and comprehensive university), academic discipline, year of study, sex, and residence status; however, random selection within strata was not documented. We therefore describe the sample conservatively as a non-probability, stratified convenience sample. The archived analytic file and source materials available for this revision do not preserve the exact month/year of data collection, the recruitment channel, the initial number approached or questionnaires distributed, reason-specific exclusion counts, or complete participant-level demographic variables. We have therefore not reconstructed these quantities retrospectively and make no claim that the final sample is representative of the wider student population. According to the archived study documentation, questionnaires meeting the prespecified quality-control criteria (obvious response errors, substantial missingness, or highly repetitive response patterns) were excluded. The archived analytic dataset contains 205 complete records; however, the initial distribution count and the number excluded under each criterion cannot be reconstructed from the surviving records. No missing-value imputation was used. Participation was voluntary and anonymous. A sensitivity analysis indicated that with N = 205 and α = 0.05, a five-predictor regression had 80% power to detect an overall effect of approximately f^2^ = 0.064 and an incremental effect of approximately f^2^ = 0.039 for an individual predictor. This calculation characterizes sensitivity of the completed regression design; it was not an a priori power calculation and does not establish adequate power for the item-level psychometric models, interactions, or conditional indirect associations.

### 2.2. Measures

#### 2.2.1. Physical Exercise

Physical exercise was assessed with the Physical Activity Rating Scale-3 (PARS-3), a Chinese-language three-item index of exercise intensity, duration, and frequency (Liang, 1994). Each component is rated on five ordered levels. The exercise-quantity score is calculated as intensity × (duration − 1) × frequency and ranges from 0 to 100. Scores ≤19 indicate low exercise, scores of 20–42 indicate moderate exercise, and scores ≥43 indicate high exercise. Because the three components are non-interchangeable and combined multiplicatively, internal consistency is not an appropriate reliability index for the composite exercise-quantity score.

#### 2.2.2. Mobile Social Media Dependence

Mobile social media dependence was assessed with Wu’s (2014) 23-item Mobile Social Media Dependence Scale for College Students, which was developed in Chinese for Chinese college students. The scale covers salience, social gain, compulsivity, withdrawal, and conflict. Items are rated from 1 (very inconsistent) to 5 (very consistent). For comparability across measures, the analysis used the item mean; higher values indicate greater dependence. Subsequent peer-reviewed studies have examined mobile social media dependence in Chinese undergraduate samples (He & Yang, 2022; Luo & Hu, 2022). He and Yang (2022) used the same 23-item instrument and reported subscale Cronbach’s α values of 0.75–0.80. These subscale estimates are reported as contextual evidence of prior use, not as direct benchmarks for the reliability of the 23-item total score. These applications provide evidence of prior research use but do not substitute for independent structural validation. Internal consistency after item-level verification in the present sample was α = 0.976. Although this value indicates high internal consistency, an alpha this high can also reflect item redundancy or narrow item-content coverage. In addition, the scale’s primary development and psychometric source is a master’s thesis rather than a large independent validation literature; these points are treated as measurement limitations rather than as evidence of superior reliability.

#### 2.2.3. Loneliness

Loneliness was assessed with the 20-item UCLA Loneliness Scale using the Chinese-language adaptation reported in the Chinese mental-health measurement literature (Russell, 1996; D. Wang, 1999). Items are rated from 1 to 4. Item-level auditing showed that the archived raw export had not been reverse-scored despite an earlier analysis-ready column being present. In accordance with the published scoring key, items 1, 5, 6, 9, 10, 15, 16, 19, and 20 were reverse-scored (5-response), and the loneliness score was calculated as the mean of the 20 keyed items. Higher values indicate greater loneliness. Internal consistency after correct scoring was α = 0.899. The exact item mapping and scoring key are reproduced in Supplementary Section S2 for transparency.

#### 2.2.4. Self-Control

Self-control was assessed with the 19-item Chinese revision of the Self-Control Scale validated for Chinese college students (Tan & Guo, 2008; Tangney et al., 2004). Items 1, 5, 11, and 14 were retained in the keyed direction; the remaining 15 items were reverse-scored (6-response). The item-mean score ranged from 1 to 5, with higher values indicating stronger self-control. Internal consistency after key verification was α = 0.753. The scoring rule is also documented in Supplementary Section S2.

### 2.3. Statistical Analysis

The item-level dataset was independently audited and analyzed in Python 3.13 (Python Software Foundation, Beaverton, OR, USA). Descriptive statistics and resampling procedures used NumPy/SciPy, and the observed-score regression models used statsmodels with ordinary least-squares equations specified to reproduce Hayes’ (2022) PROCESS Model 15. Analyses began with verification of item ranges, published reverse-scoring keys, scale construction, internal consistency, descriptive statistics, and Pearson correlations. All focal continuous variables were mean-centered before interaction terms were formed.

Because any mediation interpretation requires the proposed mediator and outcome to be empirically distinguishable, measurement diagnostics preceded pathway interpretation. A targeted item-level maximum-likelihood diagnostic CFA compared one-factor and two-factor correlation-structure solutions for the 20 loneliness and 23 dependence items using the Pearson correlation matrix. The diagnostic CFA and the polychoric/ULS sensitivity routines were implemented with custom NumPy/SciPy numerical procedures operating on item correlation matrices rather than with a dedicated SEM package. Given the ordinal response formats, the modest sample relative to the number of items, and the boundary-level factor correlation, these analyses were treated explicitly as diagnostics rather than confirmatory validation; the one- versus two-factor χ^2^ values were not used for a formal likelihood-ratio test. HTMT ratios were calculated for loneliness, dependence, and self-control as an additional discriminant-validity diagnostic (Henseler et al., 2015). To test whether the conclusion depended on treating ordinal items as continuous, a sensitivity analysis estimated pairwise polychoric item correlations, recalculated HTMT from the polychoric matrix, and fit one- and three-factor correlation-structure models using a diagonally weighted/ULS-style ordinal procedure. Because this sensitivity procedure did not implement the full threshold-based robust corrections and asymptotic weight matrix of formal WLSMV, it was also treated as a robustness diagnostic rather than a definitive ordinal CFA (C.-H. Li, 2016). A principal-axis/promax exploratory diagnostic, item-duplication audit, residual-correlation diagnostics, and 5000-resample HTMT bootstrap were examined. Detailed results are provided in Supplementary Sections S1–S10. Potential common-method variance was examined with an unrotated single-factor diagnostic across all 65 questionnaire items; this diagnostic was treated as descriptive and cannot rule out common-method bias (Podsakoff et al., 2003).

For reproducibility, the observed-score equations corresponding to Hayes’ (2022) PROCESS Model 15 were retained. The first equation regressed loneliness on physical exercise; the outcome equation regressed mobile social media dependence on exercise, loneliness, self-control, exercise × self-control, and loneliness × self-control. Conditional direct and product-of-coefficients associations were estimated at the self-control mean and at ±1 SD with 5000 percentile bootstrap resamples. Because discriminant validity was inadequate, these quantities are described as exploratory observed-score associations rather than evidence of a distinct mediation process. Heteroskedasticity was assessed using the Breusch–Pagan test, and HC3 heteroskedasticity-consistent standard errors were used for inferential interpretation. Variance-inflation factors (VIFs) were examined for multicollinearity, and the outcome model was re-estimated after excluding observations with Cook’s distance > 4/N. To address the unusually high R^2^ without creating hierarchy-sensitive drop-one comparisons in a model containing interactions, we examined three nested blocks: (i) an exercise-only model, (ii) a main-effects model adding loneliness and self-control, and (iii) the full model adding both interaction terms. These blockwise variance-decomposition analyses are descriptive because severe multicollinearity makes unique construct-specific attribution unstable. All tests were two-tailed with α = 0.05.

## 3. Results

### 3.1. Item-Level Audit, Descriptive Statistics, and Correlations

The item-level audit identified one consequential scoring problem in the initial archived scoring: the nine positively worded UCLA loneliness items had not been reverse-scored. Correcting the published key increased the loneliness internal-consistency estimate from α = 0.883 to α = 0.899 and changed the loneliness mean from the pre-audit raw-item metric to the correctly keyed metric reported below. The exercise, dependence, and self-control scoring rules were reproduced from the raw responses as specified in the Methods.

Physical exercise ranged from 0 to 100 (*M* = 23.15, SD = 23.83); 112 students (54.6%) were in the low-exercise category, 45 (22.0%) in the moderate category, and 48 (23.4%) in the high category. Dependence had *M* = 2.817 (*SD* = 1.140), correctly keyed loneliness had *M* = 2.950 (*SD* = 0.525), and self-control had *M* = 3.617 (*SD* = 0.604). Exercise was negatively correlated with dependence (*r* = −0.823) and loneliness (*r* = −0.799) and positively correlated with self-control (r = 0.723; all *p* < 0.001). Dependence correlated positively with loneliness (*r* = 0.955) and negatively with self-control (*r* = −0.860); loneliness correlated negatively with self-control (*r* = −0.838; all *p* < 0.001). Descriptive statistics and correlations are summarized in Table 1.

**Figure 2:**
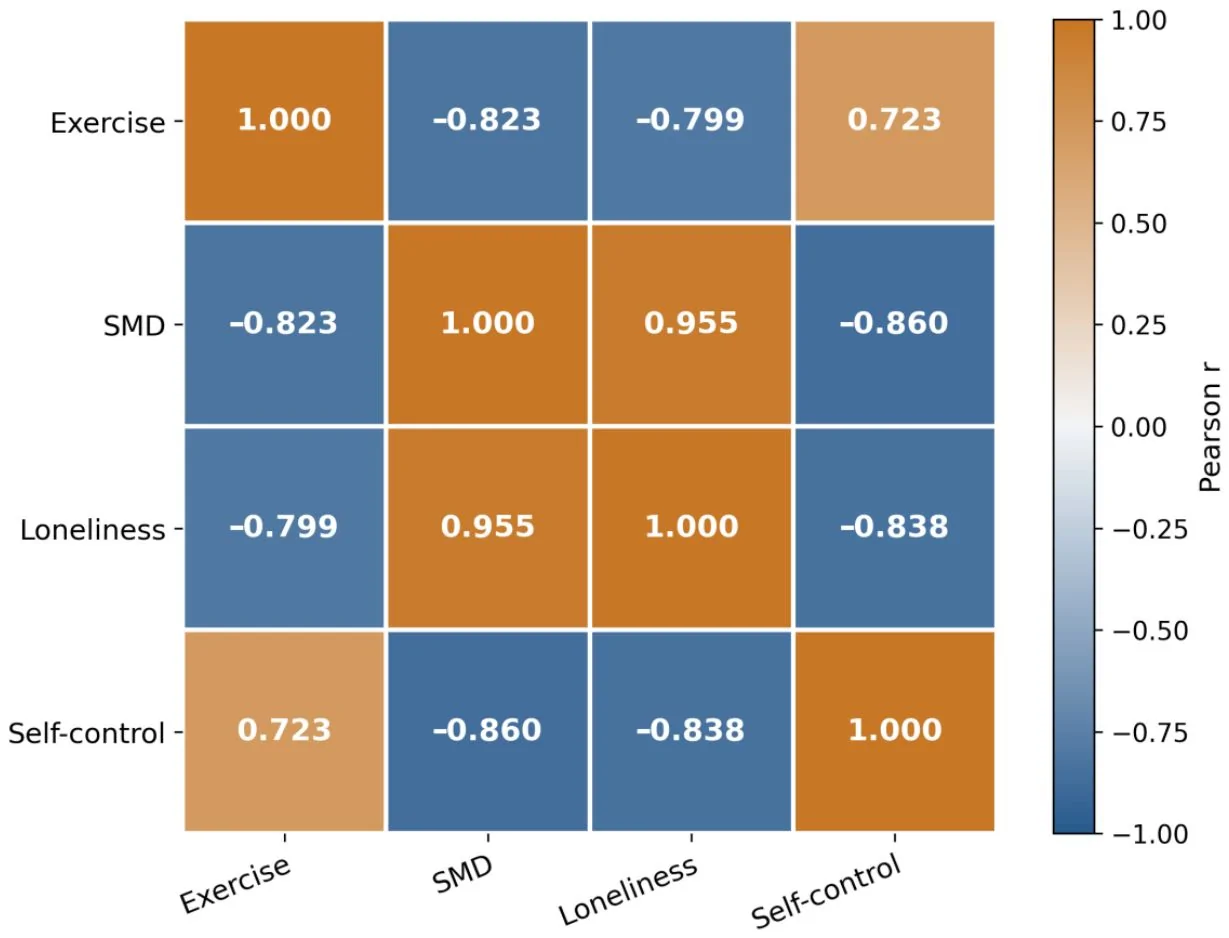
Correlation heatmap of the focal variables. Note. Cells display Pearson r values corresponding to the variables summarized in Table 1. SMD = mobile social media dependence.

**Figure 3:**
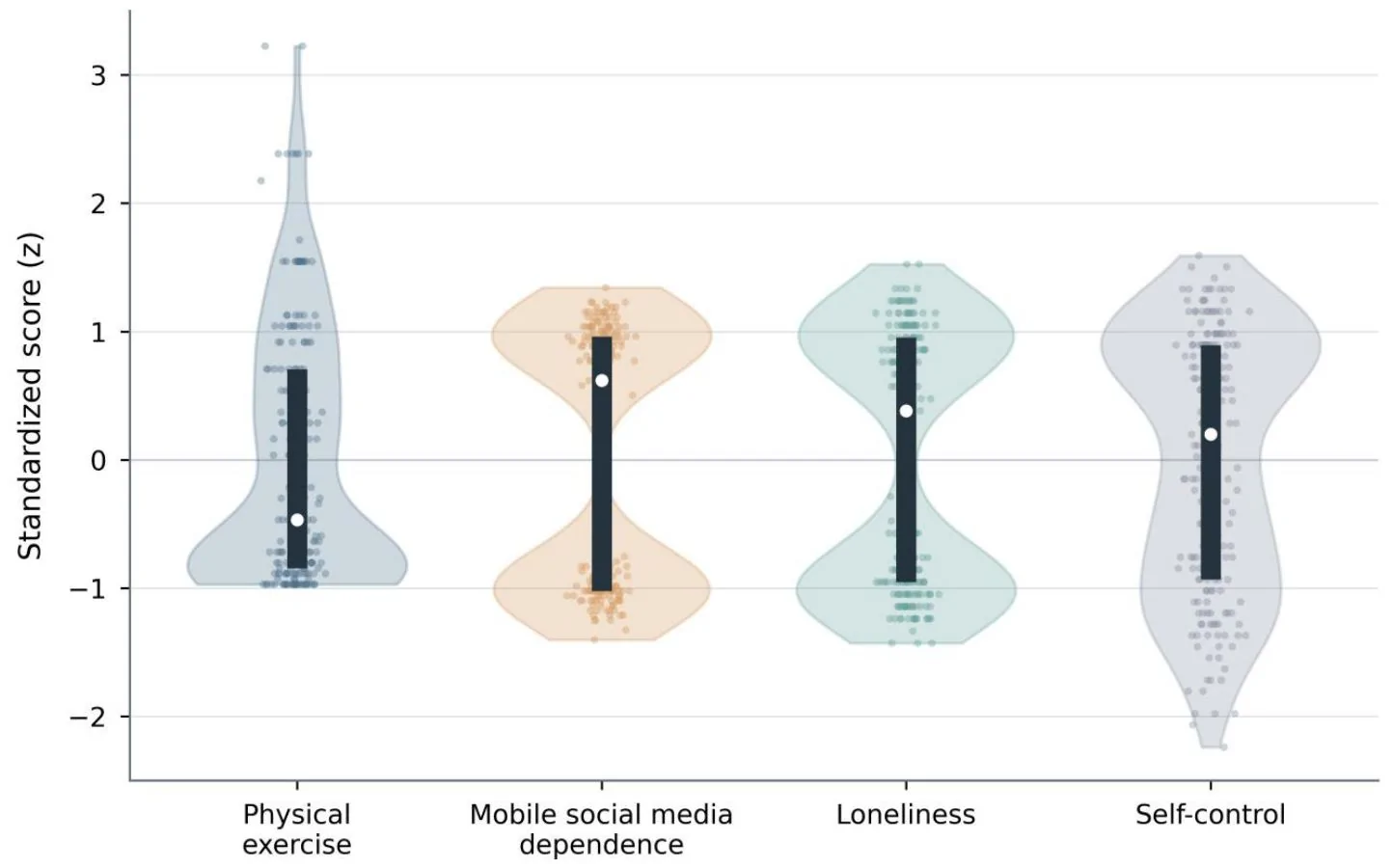
Standardized-score distributions of the focal variables. Note. Violin plots are shown on z-standardized scores to facilitate cross-variable visual comparison; overlaid points represent individual observations.

For visual inspection of the descriptive pattern, Figure 2 presents the correlation structure among the focal variables, and Figure 3 presents their standardized-score distributions.

### 3.2. Construct Validity and Method-Variance Assessment

The very high loneliness–dependence correlation prompted formal discriminant-validity diagnostics (Table 2). In the targeted diagnostic CFA, a one-factor model for the 43 loneliness and dependence items fit almost identically to a two-factor model. The one-factor solution yielded χ^2^(860) = 972.70, CFI = 0.982, TLI = 0.981, RMSEA = 0.025, and SRMR = 0.035. The two-factor solution yielded χ^2^(859) = 973.03, CFI = 0.982, TLI = 0.981, RMSEA = 0.026, and SRMR = 0.035, with the latent factor correlation estimated at approximately 0.999. Thus, separating loneliness and dependence did not produce meaningful empirical differentiation. Consistent with guidance for ordinal indicators, these results are treated as diagnostic rather than confirmatory evidence (C.-H. Li, 2016). Ordinal sensitivity analyses led to the same substantive conclusion: polychoric HTMT was 0.984 for dependence–loneliness, 0.981 for dependence–self-control, and 1.039 for loneliness–self-control. In a three-factor polychoric correlation-structure sensitivity model, the factor correlations were approximately 0.995, −0.986, and −0.995, respectively; the residual SRMR-style index was 0.05425 versus 0.05426 for the corresponding one-factor solution. Therefore, treating the items as ordinal did not materially restore factor separation (Supplementary Sections S3–S5).

Pearson-based HTMT diagnostics reached 0.994 for loneliness versus dependence, 1.024 for loneliness versus self-control, and 0.992 for dependence versus self-control. In 5000 nonparametric bootstrap samples, the corresponding 95% HTMT intervals were [0.972, 1.003], [0.998, 1.057], and [0.953, 1.010], respectively. No exact cross-construct duplicate item-response vectors were identified, and no individual cross-construct item pair reached an absolute Pearson or polychoric correlation of 0.90; the strongest cross-construct polychoric item correlation was approximately 0.805. This pattern suggests diffuse construct overlap rather than a single duplicated item column. The unrotated single-factor diagnostic produced 12 components with eigenvalues > 1, and the first component explained 43.85% of total item variance. Although no single component accounted for a majority of variance, the HTMT, CFA, ordinal sensitivity, and item-level results mean that common-method and construct-overlap explanations cannot be dismissed.

### 3.3. Exploratory Observed-Score Conditional-Process Analysis

**Figure 4:**
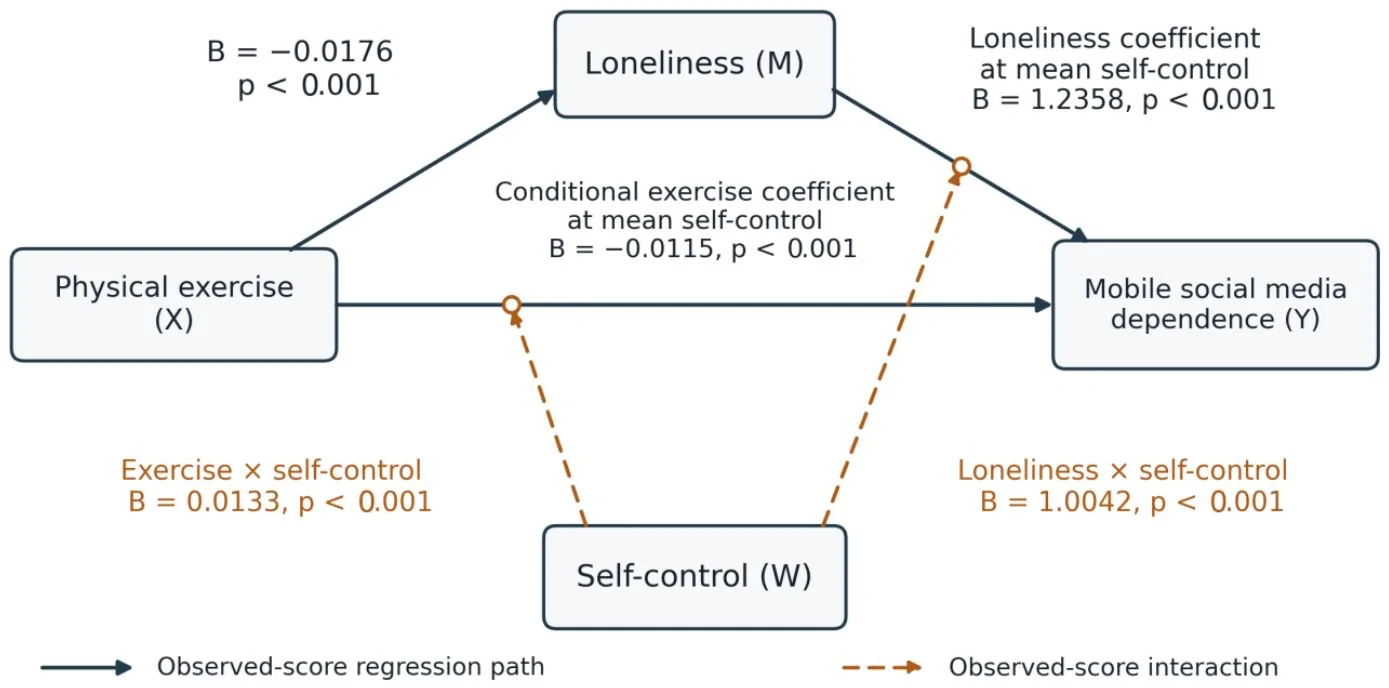
Estimated observed-score conditional-process coefficient pattern after published-key rescoring. Note. Coefficients are unstandardized. Because the measurement diagnostics indicated inadequate discriminant validity, the figure is descriptive of the fitted observed-score equations and is not evidence of a distinct mediation mechanism.

In the first-stage observed-score regression, exercise was associated with lower correctly keyed loneliness, B = −0.0176, SE = 0.0009, t(203) = −18.91, *p* < 0.001, 95% CI [−0.0194, −0.0157], R^2^ = 0.638. In the outcome model (Table 3), exercise, loneliness, self-control, and both interaction terms were statistically significant. The complete outcome model explained 94.2% of the observed dependence variance, R^2^ = 0.942, adjusted R^2^ = 0.941, F(5, 199) = 649.66, *p* < 0.001. This exceptionally high R^2^ is interpreted primarily as a warning sign of severe shared variance among the self-report measures rather than as evidence that the theoretical model has exceptional explanatory power. The resulting observed-score coefficient pattern is visualized in Figure 4.

**Figure 5:**
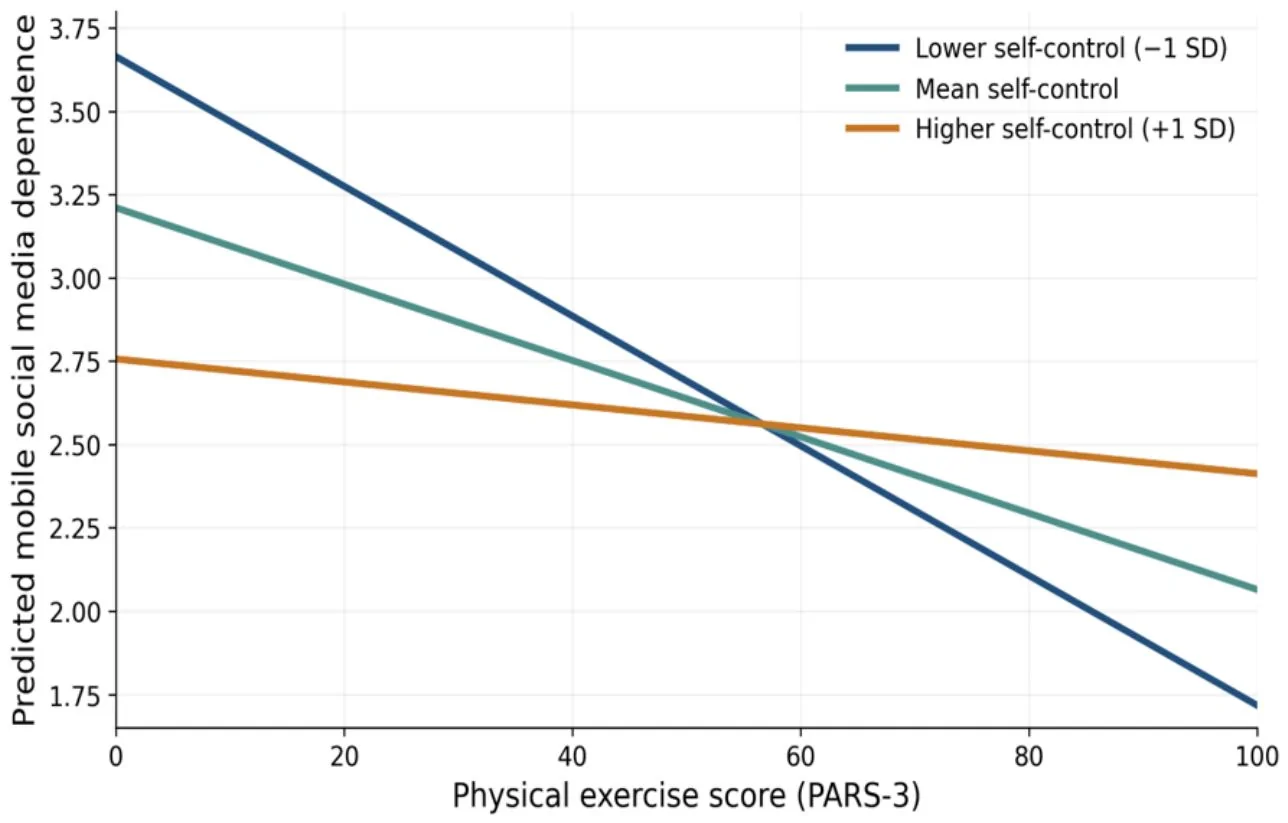
Model-implied simple slopes of physical exercise predicting mobile social media dependence at lower (−1 SD), mean, and higher (+1 SD) self-control, with loneliness held at its sample mean.

The exercise × self-control product term indicated that the negative exercise–dependence association weakened as observed self-control scores increased. At lower self-control (−1 SD), the conditional exercise coefficient was −0.0195, 95% bootstrap CI [−0.0266, −0.0115]; at the mean, it was −0.0115, 95% CI [−0.0153, −0.0073]; at higher self-control (+1 SD), it was −0.0034, 95% CI [−0.0074, 0.0006]. Thus, the simple exercise coefficient was not distinguishable from zero at the higher self-control level. This should be interpreted as a statistical interaction among observed scores; the measurement results do not establish self-control as a fully distinct psychological moderator in this sample. The corresponding simple-slope pattern is shown in Figure 5.

The product-of-coefficients association through loneliness became more negative as observed self-control scores increased (Table 4). The estimate was −0.0111, 95% bootstrap CI [−0.0167, −0.0059] at lower self-control; −0.0217, 95% CI [−0.0263, −0.0181] at the mean; and −0.0324, 95% CI [−0.0391, −0.0271] at higher self-control. The corresponding index was −0.0177, 95% bootstrap CI [−0.0244, −0.0113]. These coefficients were statistically significant and directionally stable in the reported observed-score analyses, but because loneliness and dependence were not empirically separable, they cannot be interpreted as confirmation of H2 or H4 at the construct level or as evidence of a distinct loneliness-mediated psychological mechanism.

### 3.4. Variance Decomposition, Multicollinearity, and Influence Diagnostics

An exercise-only model explained 67.8% of the observed variance in mobile social media dependence (R^2^ = 0.678; B = −0.0394, *p* < 0.001). Adding loneliness and self-control increased R^2^ to 0.930 (ΔR^2^ = 0.252; F-change = 364.15, *p* < 0.001), and adding the two interaction terms increased R^2^ to 0.942 (additional ΔR^2^ = 0.0119; F-change = 20.52, *p* < 0.001). These blockwise results show that the very high full-model R^2^ emerges in a setting of extensive shared variance rather than demonstrating unusually strong construct-specific explanatory power. The Breusch–Pagan test indicated heteroskedasticity in the full outcome model (LM = 14.76, *p* = 0.011), supporting use of HC3 standard errors. VIFs ranged from 3.75 to 6.03, indicating nontrivial multicollinearity that can destabilize partial regression coefficients. Sixteen observations exceeded the Cook’s distance heuristic of 4/N (maximum Cook’s distance = 0.124). When these observations were omitted, all five focal coefficients retained their directions, and both interaction terms remained statistically significant; the trimmed-sample outcome model had R^2^ = 0.962. These checks indicate that the coefficient pattern is not driven solely by conventional standard-error assumptions or a small set of influential cases; they do not resolve the measurement-validity problem.

## 4. Discussion

### 4.1. Interpretation and Theoretical Contribution

Three findings summarize the revised interpretation. First, exercise showed a strong inverse observed-score association with mobile social media dependence in this sample. Second, the exercise × self-control and loneliness × self-control product terms remained statistically significant and directionally stable across the reported sensitivity checks, but they should be viewed as exploratory interactions among overlapping self-report scores. Third, and most importantly, loneliness, dependence, and self-control were not sufficiently differentiated psychometrically. Consequently, neither the conditional product-of-coefficients results nor the very high model R^2^ can be used to claim a distinct mediation mechanism or exceptional explanatory power.

The inverse exercise–dependence association is compatible with several nonexclusive accounts, including time restructuring, competing non-digital reward, affect regulation, and offline engagement. However, the broader literature is not completely uniform: systematic reviews find an overall inverse pattern but emphasize heterogeneity and the limitations of predominantly cross-sectional evidence (Pirwani & Szabo, 2024, 2025), recent longitudinal work supports temporal precedence from self-reported exercise-adherence tendency to lower problematic smartphone use without establishing causality (Gai et al., 2026), and at least some university samples report no meaningful physical-activity difference by smartphone-addiction status (Kumban et al., 2025). For context, a Chinese college-student study reported a substantially smaller physical activity–problematic smartphone use correlation (r = −0.151; Zhu et al., 2023); differences in constructs and instruments limit direct comparison. The present cross-sectional data therefore cannot establish whether exercise reduces problematic use, whether problematic use displaces exercise, or whether both are influenced by unmeasured characteristics or contexts.

The role of loneliness is correspondingly more tentative than the original conceptual diagram might imply. Physical activity is not inherently social: it may occur alone or in groups, and reviews indicate that loneliness outcomes vary across interventions and may depend on deliberately created opportunities for social support and connection (Ahn et al., 2024; Kritz et al., 2026; Pels & Kleinert, 2016). Self-control has stronger prior links with problematic internet use and loneliness (Fan et al., 2022; Stavrova et al., 2022), providing a rationale for examining statistical interaction, but the present study does not establish that self-control is a distinct moderator of a distinct loneliness process. The fitted Model 15 equations are therefore best understood as an exploratory description of how observed scores co-vary, not as confirmation of H2 or H4 or of a causal psychological pathway.

### 4.2. Implications for Digital Well-Being Research

The findings are better viewed as hypothesis-generating for digital well-being research than as an intervention prescription. The most defensible substantive observation is that greater exercise co-occurred with lower mobile social media dependence in this sample. Exercise-based programs may still be promising, but the present data cannot determine whether any benefit operates through loneliness reduction, broader self-regulation, general psychosocial functioning, shared self-report variance, or other unmeasured pathways. Future intervention work should prespecify the proposed mechanism and use measures that demonstrate adequate discriminant validity before testing indirect effects.

### 4.3. Limitations and Future Research

Several limitations constrain interpretation. First, the cross-sectional design precludes temporal and causal inference and leaves reverse causation plausible. Cross-sectional mediation estimates can also be biased relative to longitudinal processes (Maxwell & Cole, 2007). Second, all focal variables were collected by self-report in the same survey. The unrotated first component explained 43.85% of item variance, and no marker variable, temporal separation, or multimethod measurement was built into the original design; common-method variance therefore remains a credible explanation for part of the covariance (Podsakoff et al., 2003). Third, the sample was a non-probability, stratified convenience sample of 205 undergraduates from three universities in one Chinese province. The archived materials do not preserve the exact data-collection month/year, recruitment channel, initial recruitment/distribution count, reason-specific exclusions, complete demographic distributions, or participant-level strata. These missing records prevent reconstruction of a complete participant-flow diagram and preclude claims of sample representativeness. Generalization beyond the observed sample should therefore be cautious.

Fourth, N = 205 is modest relative to the 43-item loneliness/dependence diagnostic CFA, the broader 62-item three-construct ordinal sensitivity analysis, and the interaction model. The post hoc regression sensitivity calculation does not resolve uncertainty about power for item-level psychometric models, interactions, or conditional product-of-coefficients associations. Fifth, discriminant validity was inadequate. The targeted diagnostic CFA, HTMT, polychoric sensitivity model, and exploratory cross-loading analyses consistently indicated that loneliness, dependence, and self-control were not sufficiently separated for strong construct-level conclusions (Henseler et al., 2015; C.-H. Li, 2016). Formal WLSMV replication in a larger independent sample remains necessary. Sixth, the scale’s original development source is a master’s thesis. Subsequent peer-reviewed applications in Chinese university samples (He & Yang, 2022; Luo & Hu, 2022) document prior use but do not substitute for independent structural validation; the present α = 0.976 also raises the possibility of item redundancy. Finally, the dependence outcome was self-reported rather than verified through device logs, and exercise was assessed with a brief self-report index rather than wearable measures. Meta-analytic evidence of discrepancies between self-reported and logged digital media use supports collecting behavioral records alongside perceived dependence, rather than treating the two as interchangeable (Parry et al., 2021). Future studies should combine preregistered scoring, independently validated measures, device-logged social media use, wearable activity, ecological momentary assessment, and longitudinal or experimental designs.

Finally, digital behavior is heterogeneous. Patterns of use, platform characteristics, and sensitivity to social feedback may have different relationships with mental health and digital engagement (da Silva Pinho et al., 2024; Sala et al., 2024). Experimental and experience-sampling studies of passive Facebook use further indicate that specific engagement patterns can affect affective well-being (Verduyn et al., 2015). A cross-cultural college-student study also examined social media use and physical activity jointly in relation to depression and anxiety (Q. Wang et al., 2025). Recent work also suggests that social support, social exclusion, anxiety, ego depletion, and physical activity may operate in more complex combinations (S. Li & Wang, 2025; J. Wang et al., 2025; Zhao & Kou, 2024). Replication should therefore test simpler, better-measured models before adding additional mediators or moderators.

## 5. Conclusions

After item-level scoring correction and statistical audit, greater physical exercise was strongly associated with lower mobile social media dependence among university students in this sample. The observed exercise–dependence association varied across levels of self-control, and the product-of-coefficients association through loneliness also varied across observed self-control scores. However, loneliness and dependence showed near-complete empirical overlap, and discriminant-validity diagnostics also indicated inadequate separation involving self-control. Accordingly, the conditional-process findings are exploratory observed-score patterns rather than evidence of a distinct loneliness-mediated mechanism, a psychologically distinct moderation process, or causal ordering. The strongest next step is independent replication with better-differentiated measures, fuller participant documentation, and longitudinal, experimental, or multimethod designs.

## Figures and Tables

**Table 1:** Descriptive statistics, reliability, and Pearson correlations.

Variable	*M*	*SD*	α	1	2	3	4	Observed Range
1. Exercise	23.151	23.832	—	—				0–100
2. SMD	2.817	1.140	0.976	−0.823 ***	—			1.217–4.348
3. Loneliness	2.950	0.525	0.899	−0.799 ***	0.955 ***	—		2.200–3.750
4. Self-control	3.617	0.604	0.753	0.723 ***	−0.860 ***	−0.838 ***	—	2.263–4.579

Note. N = 205. SMD = mobile social media dependence. α is not reported for exercise because PARS-3 is an index. Values below the diagonal are Pearson correlations. *** *p* < 0.001.

**Table 2:** Measurement and common-method diagnostics.

Diagnostic	Result	Interpretation
One-factor CFA (loneliness + dependence)	χ^2^(860) = 972.70; CFI = 0.982; TLI = 0.981; RMSEA = 0.025; SRMR = 0.035	Acceptable global fit does not demonstrate separation.
Two-factor CFA (loneliness + dependence)	χ^2^(859) = 973.03; CFI = 0.982; TLI = 0.981; RMSEA = 0.026; SRMR = 0.035; latent r ≈ 0.999	Virtually identical to one-factor solution; no meaningful discrimination.
HTMT: loneliness–dependence	0.994	Very high overlap.
HTMT: loneliness–self-control	1.024	Discriminant validity not established.
HTMT: dependence–self-control	0.992	Very high overlap.
Unrotated single-factor diagnostic	First component = 43.85%; 12 components had eigenvalues >1	Method variance cannot be ruled out; diagnostic is not definitive.

Note. CFA and ordinal factor analyses are reported as diagnostic sensitivity analyses rather than as confirmatory validation because the items are ordinal, N is modest relative to the item count, and formal WLSMV was not implemented. HTMT is interpreted as a discriminant-validity diagnostic (Henseler et al., 2015). Common-method bias cannot be ruled out from these diagnostics alone.

**Table 3:** Outcome model predicting mobile social media dependence.

Predictor	*B*	HC3 SE	*t*	*p*	95% HC3 CI
Intercept	2.9453	0.0509	57.831	<0.001	[2.8449, 3.0458]
Exercise	−0.0115	0.0021	−5.492	<0.001	[−0.0156, −0.0073]
Loneliness	1.2358	0.0955	12.942	<0.001	[1.0475, 1.4241]
Self-control	−0.4440	0.0834	−5.324	<0.001	[−0.6085, −0.2795]
Exercise × self-control	0.0133	0.0039	3.369	<0.001	[0.0055, 0.0210]
Loneliness × self-control	1.0042	0.1725	5.821	<0.001	[0.6640, 1.3444]

Note. N = 205. Predictors were mean-centered before interactions were constructed. HC3 standard errors are reported because the Breusch–Pagan test indicated heteroskedasticity.

**Table 4:** Conditional direct and product-of-coefficients associations estimated with 5000 percentile bootstrap resamples.

Self-Control Level	Value	Conditional Exercise Coefficient	95% Bootstrap CI	Conditional Indirect Association via Loneliness (95% CI)
Lower (−1 SD)	3.013	−0.0195	[−0.0266, −0.0115]	−0.0111 [−0.0167, −0.0059]
Mean	3.617	−0.0115	[−0.0153, −0.0073]	−0.0217 [−0.0263, −0.0181]
Higher (+1 SD)	4.221	−0.0034	[−0.0074, 0.0006]	−0.0324 [−0.0391, −0.0271]

Note. Estimates are based on the observed-score Model 15 equations. The “conditional indirect association” is the product of the exercise → loneliness and conditional loneliness → dependence coefficients; it is retained for reproducibility but is not interpreted as construct-level mediation because discriminant validity was inadequate.

## Data Availability

The anonymized data supporting the findings of this study are available from the corresponding author upon reasonable request, subject to institutional and participant-privacy requirements.

## Supplementary Materials

The following supporting information can be downloaded at https://www.mdpi.com/article/10.3390/bs16101908/s1: The following supporting information is provided in a separate supplementary file (Sections S1–S10): item-level construct-validity diagnostics, ordinal polychoric sensitivity analyses, bootstrap HTMT intervals, exploratory cross-loading diagnostics, duplicate-column checks, residual-correlation diagnostics, exercise-stratified sensitivity analyses, post-hoc item-removal sensitivity checks, an interpretation guide for the main manuscript, and reproducibility information for scale scoring and the nested regression checks.
