# Supplementary material for: Physical Exercise and Mobile Social Media Dependence Among University Students: Exploratory Conditional-Process Patterns Involving Loneliness and Self-Control
Source: Behav Sci (Basel). 2026 Oct 15;16(10):1908. doi: 10.3390/bs16101908 (PMC13701209; doi:10.3390/bs16101908)
Supplement: Supplementary file 1 [file behavsci-16-01908-s001.zip › behavsci-4605827-SI.pdf]

Supplementary Material: Construct-Validity and Ordinal Sensitivity Analyses

Physical Exercise and Mobile Social Media Dependence among University Students: Exploratory Conditional-Process Patterns Involving Loneliness and Self-Control

S1. Purpose and Scope

These supplementary analyses evaluate whether the severe overlap among mobile social media dependence, loneliness, and self-control can be attributed to item scoring, the treatment of ordinal items as continuous, literal duplication of item response vectors, a small number of cross-loading items, or exercise-group heterogeneity. They are intended as sensitivity analyses and do not replace a formal WLSMV replication in dedicated SEM software.

S2. Item Mapping and Scoring Audit

The archived workbook contained 65 questionnaire items. Q1–Q3 were the PARS-3 exercise items; Q4–Q23 were the 20 UCLA Loneliness Scale items; Q24–Q46 were the 23 Mobile Social Media Dependence items; and Q47–Q65 were the 19 Self-Control Scale items. UCLA items 1, 5, 6, 9, 10, 15, 16, 19, and 20 were reverse-scored (5 − response). Self-control items 1, 5, 11, and 14 were retained in the keyed direction, and the remaining items were reverse-scored (6 − response).

Construct	Items	α	M	SD
Mobile social media dependence	23	.976	2.817	1.140
Loneliness	20	.899	2.950	.525
Self-control	19	.753	3.617	.604

Note. N = 205. PARS-3 is a multiplicative index and is therefore not included in the internal-consistency table.

S3. Score Correlations, HTMT, and Bootstrap Sensitivity

Pair	Pearson r	Spearman ρ	Pearson HTMT	Bootstrap 95% CI	Polychoric HTMT	Partial r | exercise
Dependence–Loneliness	0.955	0.722	0.994	[0.972, 1.003]	0.984	0.869
Dependence–Self-control	-0.860	-0.759	0.992	[0.953, 1.010]	0.981	-0.674
Loneliness–Self-control	-0.838	-0.714	1.024	[0.998, 1.057]	1.039	-0.626

Note. Bootstrap intervals are percentile intervals from 5,000 nonparametric resamples for Pearson-based HTMT. Partial r values control the PARS-3 exercise-quantity score. The persistence of high partial Pearson correlations indicates that construct overlap is not explained solely by their shared association with exercise.

S4. Polychoric Ordinal Factor-Model Sensitivity

Diagnostic	Result
Three-factor latent correlation: Dependence–Loneliness	0.995
Three-factor latent correlation: Dependence–Self-control	-0.986
Three-factor latent correlation: Loneliness–Self-control	-0.995
Three-factor residual SRMR-style index	0.05425
One-factor residual SRMR-style index	0.05426
Largest absolute residual correlation	0.282

Note. The ordinal sensitivity model was based on a polychoric correlation matrix and a diagonally weighted/ULS-style correlation-structure fit implemented with custom NumPy/SciPy numerical routines rather than a dedicated SEM package. It was used only to test whether treating items as ordinal materially changed the substantive conclusion. It is not labeled full WLSMV because robust threshold-based mean/variance corrections and the complete asymptotic weight matrix were not implemented; no robust scaled χ^2 *comparison or formal WLSMV convergence claim is made.*

S5. Exploratory Principal-Axis/Promax Diagnostic

Intended construct	Items	Intended factor dominant	|Cross-loading| ≥ .30	|Cross-loading| ≥ .50
Dependence	23	23	0	0
Loneliness	20	6	12	11
Self-control	19	2	13	4

Note. This is an exploratory principal-axis factor diagnostic based on the polychoric matrix with promax rotation. It is not used to redefine the published scales. Dependence items formed a relatively coherent cluster, whereas many loneliness and self-control items were more strongly associated with another extracted factor than with their intended factor.

Item	Raw column	Intended construct	Intended loading	Largest cross-factor	Cross-loading
LON18	Q21	LON	-0.045	DEP	0.825
LON4	Q7	LON	-0.096	DEP	0.821
LON14	Q17	LON	0.143	DEP	0.812
LON12	Q15	LON	-0.049	DEP	0.795
SC5	Q51	SC	-0.042	DEP	0.789
LON11	Q14	LON	0.014	DEP	0.782
LON2	Q5	LON	-0.066	DEP	0.774
LON3	Q6	LON	0.013	DEP	0.768
LON13	Q16	LON	0.031	DEP	0.758
LON8	Q11	LON	0.069	DEP	0.752
LON17	Q20	LON	0.037	DEP	0.737

Item	Raw column	Intended construct	Intended loading	Largest cross-factor	Cross-loading
SC2	Q48	SC	0.072	DEP	-0.734
SC14	Q60	SC	-0.034	DEP	-0.700
SC11	Q57	SC	-0.022	DEP	-0.698
LON7	Q10	LON	0.224	DEP	0.662
SC17	Q63	SC	-0.245	DEP	-0.456
SC8	Q54	SC	-0.034	DEP	-0.452
SC7	Q53	SC	0.191	DEP	-0.422
LON1	Q4	LON	0.047	SC	0.408
SC4	Q50	SC	0.152	LON	-0.388

Note. Items are identified by scale position and archived raw-data column because item wording was not preserved in the analytic workbook. Interpretation of semantic overlap therefore requires the original questionnaire.

S6. Duplicate and Local-Misfit Audit

No exact cross-construct duplicate item-response vectors were found. No cross-construct item pair reached |Pearson r| ≥ .90 or |polychoric r| ≥ .90. The strongest cross-construct polychoric item correlation was approximately .805, indicating that the construct overlap is diffuse rather than attributable to a literal duplicated item column.

Item 1	Raw col.	Construct	Item 2	Raw col.	Construct	Polychoric r
LON4	Q7	LON	DEP15	Q38	DEP	0.805
LON4	Q7	LON	DEP23	Q46	DEP	0.787
LON14	Q17	LON	DEP22	Q45	DEP	0.784
LON4	Q7	LON	DEP17	Q40	DEP	0.776
LON4	Q7	LON	DEP11	Q34	DEP	0.773
LON13	Q16	LON	DEP22	Q45	DEP	0.769
SC5	Q51	SC	LON13	Q16	LON	0.767
SC5	Q51	SC	LON4	Q7	LON	0.760
LON8	Q11	LON	DEP8	Q31	DEP	0.755
LON4	Q7	LON	DEP4	Q27	DEP	0.754
LON13	Q16	LON	DEP6	Q29	DEP	0.752
LON13	Q16	LON	DEP18	Q41	DEP	0.752
LON13	Q16	LON	DEP11	Q34	DEP	0.751
LON4	Q7	LON	DEP16	Q39	DEP	0.750
LON8	Q11	LON	DEP22	Q45	DEP	0.750

Note. The table lists the 15 strongest cross-construct polychoric item correlations. None is sufficiently close to 1.0 to indicate an exact duplicate response column.

Item 1	C1	Item 2	C2	Observed rpoly	Model r	Residual
LON19	LON	LON15	LON	-0.283	-0.001	-0.282
LON19	LON	LON5	LON	0.264	0.007	0.257
SC15	SC	LON1	LON	0.168	-0.062	0.230
SC3	SC	LON10	LON	-0.221	0.007	-0.228
SC10	SC	SC4	SC	-0.079	0.129	-0.208
SC18	SC	SC16	SC	0.309	0.111	0.197
SC7	SC	SC3	SC	-0.002	0.192	-0.194
SC4	SC	LON15	LON	-0.186	0.003	-0.190
SC12	SC	SC9	SC	0.250	0.064	0.186
LON16	LON	LON9	LON	0.171	-0.009	0.180
LON9	LON	LON6	LON	0.174	-0.003	0.177
SC7	SC	LON5	LON	0.115	-0.061	0.176
SC1	SC	LON7	LON	0.020	-0.153	0.173
SC1	SC	LON9	LON	-0.164	0.009	-0.173
SC6	SC	LON1	LON	0.092	-0.079	0.171

Note. Residual-correlation diagnostic: 135 off-diagonal residuals had |residual| ≥ .10, 29 had |residual| ≥ .15, 5 had |residual| ≥ .20, and 2 had |residual| ≥ .25. These are residual diagnostics, not formal modification indices.

S7. Dominant-General-Factor and Exercise-Stratified Diagnostics

Eigenvalue diagnostic	Result
First polychoric eigenvalue	27.611
Second polychoric eigenvalue	1.898
First eigenvalue / second eigenvalue	14.55
Variance represented by first eigenvalue	44.53%
Variance represented by first three eigenvalues	50.56%

Note. These eigenvalue diagnostics are descriptive. The very large first eigenvalue is consistent with a dominant common dimension but does not by itself identify whether that dimension reflects substantive convergence, response style, method variance, or another source.

Exercise group	n	DEP–LON HTMT	DEP–SC HTMT	LON–SC HTMT
Low exercise (≤19)	112	0.980	0.822	0.955

Exercise group	n	DEP–LON HTMT	DEP–SC HTMT	LON–SC HTMT
Active exercise (≥20)	93	1.023	1.033	1.123

Note. Exercise stratification did not produce a consistent restoration of discriminant validity, arguing against the idea that the pooled overlap is explained only by between-group exercise differences.

S8. Post Hoc Item-Removal Sensitivity

Target	Post hoc removal required	HTMT after removal	Consequence
Dependence–Loneliness	6 loneliness items	.892	Loneliness α ≈ .739
Dependence–Self-control	8 self-control items	.885	Self-control α ≈ .694
Loneliness–Self-control	11 self-control items	.879	8 self-control items remain; α ≈ .662
All three pairs < .90	6 loneliness + 11 self-control items	.892 / .829 / .821	Substantial content loss

Note. This analysis is diagnostic only. The manuscript retains the complete published scales. Post hoc item deletion solely to achieve a conventional HTMT threshold would alter construct content and create unvalidated short forms.

S9. Interpretation for the Main Manuscript

Across continuous-score, polychoric, bootstrap, exploratory factor, duplicate-column, residual, and exercise-stratified diagnostics, the same substantive conclusion emerged: discriminant validity among the three self-report constructs was not established. The evidence does not support presenting loneliness as a distinct psychological mediator or self-control as a fully separable latent moderator in this dataset. The conditional-process results are therefore retained only as exploratory observed-score associations.

A formal WLSMV replication in lavaan or Mplus remains desirable in a substantially larger independent sample. Such replication would help determine whether the severe construct overlap observed here generalizes beyond the present dataset. The present sensitivity analyses are therefore used to delimit interpretation, not to claim formal ordinal measurement-model validation.

S10. Reproducibility Information and Incremental-Variance Diagnostics

Scale reconstruction from the archived workbook used the following verified item mapping and scoring rules: Q1–Q3 = PARS-3 exercise items; Q4–Q23 = UCLA Loneliness Scale; Q24–Q46 = Mobile Social Media Dependence Scale; and Q47–Q65 = 19-item Chinese Self-Control Scale. UCLA items 1, 5, 6, 9, 10, 15, 16, 19, and 20 were reverse-scored as 5 − response. Self-control items 1, 5, 11, and 14 were retained in the keyed direction and the remaining 15 items were reverse-scored as 6 − response. Exercise quantity was computed as intensity × (duration − 1) × frequency. Dependence, loneliness, and self-control scores were item means after keying.

To address the unusually high outcome-model R^2 and to show how explained variance accumulated across blocks, we added nested regression diagnostics using the verified observed scores. These diagnostics are descriptive rather than construct-validating because the focal predictors are highly correlated.

**Model / diagnostic**	**R^2**	**ΔR^2**	**F-change**	***p***
Exercise only	.678	—	—	< .001 for exercise coefficient
Exercise + loneliness + self-control	.930	.252	364.15	< .001
Full model + both interactions	.942	.0119	20.52	< .001

Because the full outcome model contains interactions, we do not use predictor-by-predictor drop-one R^2 values as construct-importance measures. Such comparisons are hierarchy-sensitive and are especially unstable under the severe multicollinearity observed here. The blockwise nested-model ΔR^2 values above are retained solely to show how the unusually high total R^2 accumulates across model blocks; they are not interpreted as construct-specific causal or explanatory shares. The final manuscript retains only the three nested regression blocks reported above.

Reproducibility note. The raw item-level scoring audit was performed directly from the archived questionnaire columns. The scale mapping and keying rules above allow independent reconstruction of the focal scores. The main manuscript reports the inferential model, robust standard errors, bootstrap procedures, and influence diagnostics. Formal ordinal CFA using WLSMV remains a recommended independent-replication step rather than a claim supported by the present diagnostic sensitivity analysis.
